# Why We Should Care About Regional Origins: Educational Selectivity Among Refugees and Labor Migrants in Western Europe

**DOI:** 10.3389/fsoc.2019.00039

**Published:** 2019-05-07

**Authors:** Christoph Spörlein, Cornelia Kristen

**Affiliations:** Chair for Sociology, esp. Analysis of Social Structures, University of Bamberg, Bamberg, Germany

**Keywords:** refugees, labor migrants, new immigrants, educational selectivity, regional inequality

## Abstract

Immigrant selectivity describes the notion that migrants are not a random sample of the population at origin, but differ in certain traits such as educational attainment from individuals who stay behind. In this article, we move away from group-level descriptions of educational selectivity and measure it as an individual's relative position in the age- and gender-specific educational distribution of the country of origin. We describe the extent of educational selectivity for a selection of Western European destinations as well as a selection of origin groups ranging from recent refugee to labor migrant populations. By contrasting refugees to labor migrants, we address longstanding assumptions about typical differences in the degree of selectivity between different types of immigrants. According to our findings, there are few and only minor differences between refugee and labor migrants. However, these differences vary; and there are labor migrant groups that score similar or lower on selectivity than do the refugees covered in this study. Selectivity differences between refugees and labor migrants therefore seem less prominent than arguments in the literature suggest. Another key finding is that every origin group is composed of varying proportions of positively and negatively selected individuals. In most cases, the origin groups cover the whole spectrum of selectivity, so that characterizing them as either predominantly positively or negatively selected does not seem adequate. Furthermore, we show that using country-level educational distributions as opposed to sub-national regional-level distributions can lead to inaccurate measurements of educational selectivity. This problem does not occur universally, but only under certain conditions. That is, when high levels of outmigration from sub-national regions in which economic opportunities are considerably above or below the country average, measurement inaccuracy exceeds ignorable levels. In instances where researchers are not able to use sub-national regional measures, we provide them with practical guidance in the form of pre-trained machine-learning tools to assess the direction and the extent of the measurement inaccuracy that results from relying on country-level as opposed to sub-national regional-level educational distributions.

## Introduction

Individuals who leave their country of origin rarely represent a cross-section of the origin population, but differ in important characteristics from individuals who remain in their home country. Among the most frequently described features are age and gender (Lindstrom and López Ramírez, 2010), health (Weeks et al., 1999; Lu, 2008; Ro et al., 2016), ambition and risk-seeking behaviors (Bonin et al., 2006; Van Dalen and Henkens, 2012) and, crucially, educational attainment (Chiquiar and Hanson, 2005; Feliciano, 2005, 2008; Ibarraran and Lubotsky, 2007; McKenzie and Rapoport, 2010; Grogger and Hanson, 2011; Belot and Hatton, 2012; Ichou, 2014; Lessard-Phillips et al., 2014; Rendall and Parker, 2014; Spörlein, 2014; Spörlein and Kristen, 2018). More than half a century ago, Everett S. Lee succinctly put this notion of immigrant selectivity in his assertion that migrants are “not a random sample of the population at origin” (Lee, 1966, p. 56).

For decades, the nature of this non-random selection of migrants has been subject of debates with some researchers arguing that immigrants are negatively selected in terms of educational attainment while others argue to the contrary. Usually, these assessments are qualified with regard to certain conditions that are expected to shape the degree of educational selectivity, for example, with respect to the type of migration (e.g., Borjas, 1987; Chiswick, 1999), economic and other macro-level conditions (e.g., Jasso and Rosenzweig, 1990; Cobb-Clark, 1993; Van Tubergen et al., 2004; Levels et al., 2008; Dronkers and de Heus, 2010; Spörlein and van Tubergen, 2014) or characteristics that are seen as typical for immigrants such as their ambition or drive to succeed (e.g., Feliciano, 2005; Ichou, 2014). No matter of the argument brought forward, there seems to be a unifying feature to these considerations. That is, educational profiles are seen as indicative for immigrants' integration potential and consequently for the prospects of a successful incorporation into the receiving society (Portes and Rumbaut, 1996; Chiswick, 1999; Van Tubergen et al., 2004; Levels et al., 2008; Dronkers and de Heus, 2010; Spörlein and van Tubergen, 2014). Empirical studies on the consequences of educational selectivity, for example, highlight its relevance for learning the destination language with more positively selected individuals acquiring language skills faster (Spörlein and Kristen, 2018). Studies on the second generation, to date, have mostly examined whether educational selectivity in the parental generation affects the education of their children (e.g., Feliciano, 2005, 2008; Ichou, 2014; Feliciano and Lanuza, 2017; Van de Werfhorst and Heath, 2018). In most cases, the findings confirm that a positive selection in the parental generation fosters children's educational attainment. Yet others have investigated the consequences for immigrants' labor market performance (e.g., Picot et al., 2016).

In this study, we aim at describing educational selectivity for a range of immigrant groups who recently came to Western Europe. We use the geographical term rather broadly to refer to a selection of European countries that in recent decades became important destinations for immigrants. In the immediate past, some of these countries even turned into crucial receiving societies worldwide, with immigration rates surpassing those of classic destinations (OECD, 2016). Based on the available data sources, we are able to study immigrant selectivity in England, Germany, Ireland, the Netherlands and Spain.

In combination with its increase in size, Western Europe's migrant population became much more diverse over time. It now covers individuals of many different origins who migrated for a variety of reasons and under different legal circumstances. In our description of educational selectivity, we focus on refugees from Syria and other conflict regions in South Asia (Afghanistan) and the Middle East (Iraq). We contrast their educational profiles with those of labor migrants and their families from a variety of origins. The available data allows distinguishing between labor migrants from Eastern Europe (Bulgaria, Poland, Romania and the Ukraine^1^) as well as from a range of so-called third countries (i.e., non-EU member states). These countries are located in Africa (Morocco), the Middle East (Turkey), South Asia (Pakistan), and Latin America (Argentina, Bolivia, Brazil, Colombia, Cuba, Ecuador, Peru, and Venezuela). By comparing refugees to labor migrants and their family members, it is possible to assess differences in the degree of selectivity between different types of migrants. Specifically, we can address the longstanding assumption that refugees are less positively selected compared to economic migrants (Chiswick, 1999).

For this descriptive undertaking, we build upon and go beyond prior measurement approaches to selectivity^2^. Much of the literature frames selectivity from the perspective of the receiving society rather than from that of the country of origin. In fact, most empirical studies on migrant selectivity do not rely on data for non-migrants in the origin country. Instead, they refer to macro-level characteristics of the country of origin and/or destination, such as cross-country differences in the level of economic development (e.g., Cobb-Clark, 1993; Levels et al., 2008) or net earning differentials between migrants and majority members in the destination country (e.g., Borjas, 1987). Even studies that explicitly consider the country of origin as the point of comparison are frequently limited by their focus on group-level processes. In this perspective, selectivity is treated as a characteristic of an immigrant group as a whole rather than as an individual-level attribute (e.g., Borjas, 1987; Feliciano, 2005; Ibarraran and Lubotsky, 2007; McKenzie and Rapoport, 2010; Rendall and Parker, 2014; Van de Werfhorst et al., 2014; Ro et al., 2016; Van de Werfhorst and Heath, 2018). This group-level characterization of immigrant selectivity perpetuates a narrative according to which some migrant groups are drawn from the higher end of the educational distribution, whereas the opposite is true for other groups. However, using a measure of selectivity at the group level obscures that immigrants of the same origin may have acquired more or less education than indicated by the overall group value.

Moving away from group-based definitions of selectivity toward a definition at the individual level and therefore toward a more direct conceptualization of selectivity, Ichou (2014) introduced a measure that indicates the individual migrant's relative position in the educational distribution of the country of origin. This individual-level perspective explicitly acknowledges that an origin group can consist of varying shares of both positively and negatively selected individuals. In fact, migrant groups often consist of individuals covering the whole selectivity spectrum rather than of individuals concentrating on one end or around a certain value of that spectrum.

In this paper, we further refine Ichou (2014) individual-level approach by describing educational selectivity relative to the population in migrants' sub-national region of origin as opposed to the whole population in the country of origin. Our focus on sub-national regional selectivity is driven by two considerations. First, there is substantive variation in educational distributions within origin countries and a narrow focus on country-level distributions obscures this sub-national regional heterogeneity. Second, there are historic cases of migration flows of individuals who had distinct educational profiles and came from confined regions of their origin country rather than from all over the country. If these kinds of emigration patterns are accompanied by sub-national regional variation in educational distributions, selectivity measures that consider a country as a whole—at either the group or the individual level—will be inaccurate. Thus far, this regional nuance has been largely absent from the literature. For ease of presentation, throughout this article, we refer to the sub-national regional level as “regional level.”

Our descriptive undertaking entails the attempt to assess and quantify the inaccuracy that is introduced by relying on national averages instead of more fine-grained distributions at the regional level. Starting with the description of the inaccuracy for a range of immigrant groups in different destinations, we intend to address a selection of macro-level conditions associated with the degree of inaccuracy. Moreover, we use machine-learning techniques to estimate its direction and extent. The application allows for an identification of origin countries, in which potential distortions introduced by relying on national level rather than on regional data are likely to occur.

## Why Relative Education Matters (in Addition to Absolute Education)

Readers may wonder whether information on relative education in terms of the position migrants occupy in the educational distribution of their origin country provides additional insights compared to the commonly established strategy of focusing on absolute educational attainment. At least three arguments seem relevant in this context.

First, educational attainment can be a sometimes-noisy indicator of skill levels, which is not easily comparable across countries. That is, two individuals from two different countries who have acquired the same level of absolute education may not necessarily have acquired the same level of skills. One of the reasons for potential discrepancies in this regard is that educational systems differ in their capabilities of conveying competences.

Second, the value a certain degree has in a society varies with the prevalence of this degree. As countries differ in their economic development and, relatedly, in how far the educational expansion has gone, having acquired a medium or higher degree may mean very different things across contexts. This consideration seems especially relevant for migrants from less developed countries who settle in modern, highly industrialized societies.

Third, an individual's relative education might represent a range of latent, usually unmeasured characteristics and resources that are expected to influence immigrants' incorporation into the receiving societies (Spörlein and Kristen, 2018). These unmeasured traits include migrants' motivation and their drive to succeed (Feliciano, 2005). Selectivity may also stand for other skills such as cognitive competences (Chiswick and Miller, 2001) or other academically useful resources (Feliciano, 2008; Ichou, 2014). In addition, the status position immigrants held prior to migration may continue to be relevant for their perceptions and behaviors, especially when the actual absolute status position in the destination country is lower than that held in the origin country (Ichou, 2014; Feliciano and Lanuza, 2017). In these instances, individuals drawn from higher positions in the origin country's status hierarchy likely experience status inconsistency. This perceived mismatch could be a motivating factor that triggers investments aimed at improving upon lower post-migration status. Considering migrants' relative education may thus allow capturing characteristics typical for a higher status position that would go unnoticed when focusing exclusively on absolute education.

Taken together, we argue that combining information on absolute education with a relative measure of educational attainment that records the individual's position in the educational hierarchy of the origin country allows for a more accurate description of the educational composition of migrant populations. In addition, by considering relative education, it is possible to address attributes and characteristics that are often overlooked or not covered in data collections, but which nevertheless may matter for migrants' and their children's prospects in the destination country.

## Selectivity Profiles of Refugees and Labor Migrants

The notion that labor migrants and refugees differ in their selectivity profiles was put forward in two major contributions in economics (i.e., Borjas, 1987; Chiswick, 1999), which became an important source also for the sociological literature. Notably, Chiswick (1999, p. 181) characterized labor or economic migrants as “tending on average to be more able, ambitious, aggressive, entrepreneurial, or otherwise more favorably selected than similar individuals who choose to remain in their country of origin.” They are contrasted with individuals “for whom other motives are important such as tied movers, refugees, and ideological migrants” (Chiswick, 1999, p. 181). According to his reasoning, the difference between labor migrants and refugees boils down to the motive to migrate. That is, individuals who strive to improve their economic situation should be more positively selected than those who respond primarily to push-factors of migration such as the refugees covered in our study (i.e., Afghans, Iraqis, and Syrians) who mostly have left their origin countries due to violent conflict and war.

Borjas (1987) provides a different view, which is influenced by the refugee movements during the Cold War. He expects that a communist takeover and the subsequent wealth redistribution negatively affects the more entrepreneurial-minded segments of the local population and motivate them to emigrate (Borjas, 1987, p. 534). Hence, for this specific historic case of refugee movement, Borjas predicted a positive selection of refugees; at least, he did not assume that they differ from labor migrants.

The literature on immigrant selectivity is dominated by the notion of migration motives being an important reason for selectivity differences between labor migrants and refugees. Empirical studies investigating his idea can be grouped into two strands. One strand is addressing the extent and direction of selectivity (e.g., Jasso et al., 2004; Feliciano, 2005; Grogger and Hanson, 2011; Lessard-Phillips et al., 2014); the other strand is using selectivity arguments to study differences in integration outcomes (e.g., Van Tubergen et al., 2004; Levels et al., 2008; Dronkers and de Heus, 2010; Spörlein and van Tubergen, 2014).

Regarding the first strand, Jasso et al. (2004) address immigrants' health and report particularly negative health selectivity among refugees. This reasoning could also be relevant for the refugees covered in our study, who, in addition to their experience of war and conflict, often fled under dangerous and potentially traumatizing conditions.

Moving to educational selectivity, Feliciano (2005) study provides a contrasting picture to the assumption of negative selectivity among refugees. She shows that virtually all large origin groups present in the United States are on average positively selected, including migrant groups, in which political refugees (e.g., from Cuba or Iran) play an important role. However, in contrast to our study, the refugees covered in her analyses mostly have not been leaving their home countries during a war.

Extending the scope of destination countries to other English-speaking and European societies, Grogger and Hanson (2011) provide indirect evidence for the idea that refugees are negatively selected by showing that migrants who arrive in countries with more liberal refugee and asylum policies tend to be less skilled. Lessard-Phillips et al. (2014) pursue a similar route by comparing selectivity profiles of immigrants in countries with small refugee populations to selectivity profiles of immigrants in countries with larger refugee populations. Their results are ambiguous for two important host countries for refugees, namely, Finland and Sweden. For Finland, they report predominantly positive selectivity patterns; for Sweden, the results point to a slightly positive or a negative selectivity.

The second strand of research uses selectivity arguments to inform analyses of differences in integration outcomes across immigrant groups, often from a cross-national perspective. This literature frequently refers to the reasoning of Borjas (1987) and Chiswick (1999) and points to macro-level indicators that are expected to reflect selectivity differences between migrant populations. Refugee streams, for example, are approximated by the degree of political suppression in the origin countries. Immigrants from these countries should be less positively selected and therefore less successful in their host societies. This indirect approach to immigrant selectivity is accompanied by mixed evidence. Migrants from countries with high levels of political suppression are less likely to be employed (Van Tubergen et al., 2004), and their children score lower in math (Levels et al., 2008). At the same time, political suppression seems to be unrelated to migrants' occupational status (Spörlein and van Tubergen, 2014) and to their offspring's science test scores (Dronkers and de Heus, 2010).

To summarize, both strands of research rely on group-level characterizations of immigrant populations as either positively or negatively selected. They use a range of different measures of selectivity of which most are indirect and based on macro-level characteristics. Overall, there seems to be inconsistent evidence and little agreement in the empirical description of selectivity of refugee populations and of the differences to labor migrants. In the following, we provide an overview of measurement approaches and address potential solutions to the problem of using aggregate and indirect methods to describe and analyze immigrant selectivity.

## Measuring Educational Selectivity

Much of the existing literature frames selectivity from the perspective of the destination countries. A prominent example refers to the aftermath of the period of labor recruitment in Western Europe in the 1960s, when many immigrants worked in low-skill jobs. Since then, it was often assumed that these immigrants were negatively selected in terms of their human capital. This assessment was usually made in comparison to the majority population in the destination country rather than in comparison to the populations in the countries of origin. However, for a sending country in which the average level of education is lower, a medium educational degree is relatively more valuable than it is in a context in which the average level of education is higher and where most individuals complete at least a medium degree. In other words, immigrants who do not have a high education according to the standards in the destination country may nonetheless be quite selective relative to the general population in their home countries (Lieberson, 1980, p. 214).

Still, most empirical studies on selectivity do not rely on data for non-migrants in the country of origin. Instead, they attempt to capture selectivity by referring to macro-level attributes of the country of origin and/or destination. Typical examples of this approach include the distance between the origin and the destination country, income inequality or relative levels of economic development (e.g., Borjas, 1987; Jasso and Rosenzweig, 1990; Cobb-Clark, 1993; Van Tubergen et al., 2004; Levels et al., 2008; Dronkers and de Heus, 2010; Spörlein, 2014; Spörlein and van Tubergen, 2014). Indicators of this kind provide indirect approximations of educational selectivity. More direct measures, in contrast, compare migrants with those who remain in the country of origin (e.g., Feliciano, 2005; Grogger and Hanson, 2011; Belot and Hatton, 2012; Lessard-Phillips et al., 2014). Because they rely on databases that provide information about the populations who did not emigrate, these measures are better suited to capturing differences between immigrants and the population in the country of origin.

Even studies that explicitly consider the country of origin as the point of comparison are frequently limited in that they treat selectivity as a characteristic of an immigrant group as a whole rather than as an individual-level characteristic (e.g., Borjas, 1987; Feliciano, 2005; Ibarraran and Lubotsky, 2007; McKenzie and Rapoport, 2010; Rendall and Parker, 2014; Van de Werfhorst et al., 2014; Ro et al., 2016; Van de Werfhorst and Heath, 2018). Using a measure of selectivity at the group level obscures that migrants from the same country of origin may have acquired more or less education than indicated by this overall group value. As many immigrant groups will consist of varying shares of positively and negatively selected individuals, these measures yield rough and sometimes overly general assessments. They are especially problematic for groups with highly dispersed or with non-normal educational distributions of educational attainment. For example, consider a bimodal distribution in which a substantial share of the population has received little education while another substantial share is well educated. In this case, an average selectivity measure at the group level will misrepresent the group's overall educational composition. As we will demonstrate later on, distributions of this kind are not exceptional, but occur rather frequently.

One way to avoid these problems is to move away from group-based definitions of selectivity toward a definition at the individual level and therefore toward a more direct conceptualization. Along these lines, Ichou (2014) recently introduced a selectivity measure that indicates the individual migrant's relative position in the educational distribution of the country of origin. We create this measure by first assigning each immigrant to the appropriate educational distribution in the country of origin and thereby allowing a comparison to individuals of the same age and gender who did not migrate. In a next step, we calculate each individual's position in the relevant educational distribution. This individual-level approach not only goes beyond overly general findings that some groups are negatively selected while the reverse is true for others, but it also acknowledges that an origin group is composed of varying proportions of both positively and negatively selected individuals.

Although the implementation of a direct individual-level measure of selectivity reflects a significant step forward, its application may not necessarily take into account variation in educational distributions within origin countries. At the same time, within-country differences in educational distributions are quite frequently substantial. They are related to differences in the socio-economic structure of the population; and they can be a result of regional disparities in educational opportunities (e.g., regarding the quality of educational input or the distances to different kinds of schools; Ulubaşoglu and Cardak, 2007; Qian and Smyth, 2008). In addition, there are cases of migration flows from confined regions of their country of origin. A prominent example refers to Turkish labor migrants who arrived as “guest workers” in different Western European destinations between 1961 and 1974; they originated mostly from rural areas in middle Anatolia (Guveli et al., 2016). Another important example concerns the migration stream between Mexico and the United States, which is dominated by Mexicans from rural areas (Ibarraran and Lubotsky, 2007; Rendall and Parker, 2014). In general, regional variation in outmigration rates seems to be greater in countries, in which the opportunity structure substantially differs across regions (Rathor and Premi, 1986; Portnov, 1999; Heidenreich and Wunder, 2008; Enflo and Rosés, 2014). Given this typical combination of higher rates of outmigration from regions that tend to be further away from the national average, the construction of selectivity profiles based on national averages seems problematic. It can yield inaccurate assessments of the degree of educational selectivity.

## Data and Methods

### Destination and Origin Country Data on Educational Attainment

The envisaged empirical analyses make large demands on the data sources, both for the countries of origin and destination.

To our knowledge, in the Western European context, currently only three data sources include information on immigrants' regional origin. The empirical account, therefore, is limited to the receiving societies comprised in these data sets. These countries are Germany, England, Ireland, the Netherlands and Spain.

The first data set is the *IAB-BAMF-GSOEP Survey of Refugees in Germany* (IBS-RS.) With a total sample of roughly 4,500 individuals aged 18 and older, it covers the largest refugee origin populations who arrived in Western Europe between 2013 and 2016 (Brücker et al., 2016). Although Germany is only one receiving context for refugees, it is by far the largest recipient with more than half of the total refugee population heading for Europe eventually settling there (Bundesministerium des Inneren., 2016; Eurostat, 2016).

Second, information on labor migrants comes from the first wave of the two-wave panel SCIP (*Socio-Cultural Integration Processes among New Immigrants in Europe*; Diehl et al., 2015; Gresser and Schacht, 2015). The data covers recent migrants in England, Germany, Ireland and the Netherlands. Around 8,000 individuals, aged 18 to 60, were surveyed in 2010/2011. They have been staying in the respective destination country up to 18 months. The origin groups included in SCIP come from countries with which the destinations have shared a history of labor recruitment (i.e., Turks in Germany and the Netherlands, Moroccans in the Netherlands) or have had former colonial ties (i.e., Pakistanis in England). In addition, the data reflect recent flows of labor migrants from Eastern Europe (i.e., Poles in all 4 countries).

Third, we include *The National Immigrant Survey of Spain* (ENI) from 2008 as an additional source of data on labor migrants (Reher and Requena, 2009). It covers around 15,500 foreign-born immigrants 16 years of age and older who have lived in Spain between 1 and 8 years. We exclude immigrants who completed their education in Spain. During the period that is represented in this dataset (1998–2006), Spain saw a massive increase in migrant stock, which rose from around 2 to almost 10 percent. The origin group composition reflects the immigration patterns of this period, in which immigration from Latin America, North Africa and Eastern Europe dominated (Reher and Silvestre, 2009).

Table 1 provides information on the various data sources including a brief description of the sampling procedures as well as a list of the various migrant groups covered. The analyses are confined to origin groups with at least 100 cases. Distributions of the key indicators are depicted in Tables S1, S2.

**Table 1:** Destination country data.

**Data set**	**Duration of stay**	**Period of immigration**	**Sampling**	**Destination country**	**Immigrant groups**
IAB-BAMF-GSOEP Survey of Refugees in Germany (IBS-RS)	New immigrants: Up to 3 years (>90 percent no longer than 2 years)	2013–2016	Random sample of Central Register of Foreigners (AZR); oversampling of groups who had a higher likelihood of staying (i.e., Afghans, Iraqis and Syrians), women and individuals older than 30	Germany	Refugees from - South Asia/Middle East (Afghanistan: *n* = 460, Iraq: *n* = 485, Syria: *n* = 2,046)
Socio-Cultural Integration Processes among New Immigrants in Europe (SCIP)	New immigrants: Up to 18 months	2008–2010	Respondent-driven sampling in London (RDS)	England	Labor migrants from - South Asia (Pakistan: *n* = 634) - Eastern Europe (Poland: *n* = 479)
			Respondent-driven sampling in Dublin (RDS)	Ireland	Eastern Europe (Poland: *n* = 982)
			Stratified random sample from register data in five large cities	Germany	Eastern Europe (Poland: *n* = 1,272) - Middle East (Turkey: *n* = 981)
			Stratified random sample from national register data	Netherlands	- Africa (Morocco: *n* = 221) - Eastern Europe (Bulgaria: *n* = 315, Poland = *n* = 372) - Middle East (Turkey: *n* = 562)
The National Immigrant Survey of Spain (ENI)	New immigrants and immigrants with longer durations of stay: At least 1 year up to 8 years	1998–2006	Random household sample of foreign-born residents from register data	Spain	Labor migrants from - Africa (Morocco: *n* = 404) - Eastern Europe (Bulgaria: *n* = 260, Romania: *n* = 1,109, Ukraine: *n* = 163) - Latin America (Argentina: *n* = 389, Bolivia: *n* = 295, Brazil: *n* = 158, Colombia: *n* = 641, Cuba: *n* = 100, Ecuador: *n* = 932, Peru: *n* = 139, Venezuela: *n* = 113)

*All data sets are accessible to researchers*.

Table 1 reveals that information on refugees is available only for Germany (IBS-RS), whereas labor migrants can be studied in all five destinations. In principle, both the SCIP and ENI—the two data sources we rely on to study labor migrants—could also include refugees. Using information on migration motives, it turned out that < 0.5% of SCIP respondents indicated migrating for political reasons, whereas none of the respondents did so in the Spanish data. In addition, none of the origin countries in these two data sets was engaged in a war or other forms of major violent conflicts during the respective immigration periods, which might have contributed to sizable refugee streams. Taking together the negligible numbers of migrants who indicated political migration motives and the absence of violent conflicts leads us to conclude that the SCIP and ENI data provide a solid foundation to study labor migrants and their family members.

Moreover, two of the three data sets focus exclusively on new immigrants (IBS-RS and SCIP), while the third (ENI) includes recently arrived individuals as well as immigrants with longer durations of stay. Additional variation is introduced with regard to the immigration periods covered by the different sources. Finally, given that new migrant populations are often difficult to sample because sampling frames are not always available, sampling strategies differ across and partly also within the data sets depending on the destination country and the immigrant groups considered. For these reasons, we do not claim to come up with a fully comparable empirical account across migrant groups and destinations.

To consider region-, gender- and age-specific educational distributions, we rely on a variety of data sources. The regionalized and country-level distributions are constructed based on micro data from the IPUMS-International project (Integrated Public Use Microdata Series International), which collects and harmonizes census data from a host of different countries, the UNICEF-MICS (Unicef Multiple Indicator Survey), the DHS (US Aid Demographic and Health Survey) Program, the EU-LFS (European Labor Force Survey) and the Turkish Statistical Institute. Table 2 lists the data sets for the different countries of origin. Whenever possible, regional classifications are based on the first-level administrative divisions published by the International Organization for Standardization (ISO). For six of the groups, the origin data required us to aggregate administrative divisions to achieve comparability (see Table 2 for more information).

**Table 2:** Origin country data on educational attainment.

**Country**	**Year**	**Source**	**Sample size (in thousands)**	***N* administrative units**
Afghanistan	2011	MICS	86	8^a^
Argentina	2010	IPUMS-I	3.937	24
Bolivia	2004	DHS	17	9
Brazil	2010	IPUMS-I	9.693	6^a^
Bulgaria	2009	EU-LFS	14	6
Colombia	2005	IPUMS-I	3.643	11^a^
Cuba	2006	MICS	27	4
Ecuador	2010	IPUMS-I	1.269	7^a^
Iraq	2011	MICS	238	18
Morocco	2004	IPUMS-I	1483	14^a^
Pakistan	2013	MICS	17	6
Peru	2007	IPUMS-I	2.585	25
Poland	2011	IPUMS-I	3.194	16
Romania	2011	IPUMS-I	1.992	42
Syria	2006	MICS	96	14
Turkey	2011	TurkStat	54.000	82
Ukraine	2005	MICS	29	5^a^
Venezuela	2000	IPUMS-I	2.306	22

a *Data based on an aggregated version of the country's administrative division (aggregation to achieve correspondence in the measures of regional origin between origin and destination data sources); DHS, US Aid Demographic and Health Survey; EU-LFS, European Labor Force Survey; IPUMS-I, Integrated Public Use Microdata Series International; MICS, Unicef Multiple Indicator Cluster Survey; TurkStat, Turkish Statistical Institute. All data sets are accessible to researchers*.

### The Selectivity Measure: Relative Education

In the origin and destination country data likewise, educational attainment is measured by four categories based on a variant of the *International Standard Classification of Education* (ISCED-97). We distinguish between “primary completed” (ISCED 0, 1), “some secondary” (ISCED 2), “secondary completed” (ISCED 3, 4), and “tertiary completed” (ISCED 5, 6). Reducing the information on educational attainment by combining categories is unavoidable given the cross-national comparative scope of the analyses and the use of many different data sources.

For the refugee populations in the IBS-RS data, educational attainment was measured in more detail. Consequently, for this group we are able to provide a description of selectivity based on the ISCED-97 classification without collapsing ISCED 0 and 1 as well as ISCED 3 and 4 into one category. We will present this more detailed specification together with the less detailed measure on which we have to rely for all other groups. This comparison allows illustrating the impact ostensibly minor changes in core measurements can have for the assessment of selectivity.

The coding of immigrants' education in the destination country data may be less problematic than it is in other instances. This is because the three surveys IBS-R, SCIP, and ENI explicitly address immigrants and therefore do not implement measures that are designed to reflect the degrees obtained in the destination country. Quite to the contrary, both the IBS-R and the SCIP data, which target recently arrived immigrants, ask for the educational degrees that are typical for each of the origin countries. The Spanish data set ENI includes information on the highest level of studies acquired in the country of origin. It is measured with an open question. This proceeding does not seem to force respondents either to assign their qualification to a degree that is typical for Spain.

In the origin country data, age is categorized into ten 5-year units covering individuals aged 15–64 years. To give the reader a sense of the number of educational distributions that are taken into account, consider an exemplary origin country with 10 regions. Then for each region, we construct 2 [gender categories] ^*^ 10 [age categories] = 20 reference distributions. For the 10 regions, these distributions add up to 200 reference distributions.

Combining destination with origin data enables us to create an individual-level measure of selectivity by (1) assigning each immigrant to the appropriate gender- and age-specific educational distribution in the country of origin and subsequently (2) calculating his or her relative position in the reference distribution. The resulting index of selectivity represents the percentage of individuals with a lower level of educational attainment compared to the individual migrant plus half the percentage of individuals with the same level of education; this calculation positions the immigrant in the center of the respective educational category. Put differently, this measures records an individual's quantile position in the origin country's gender- and age-specific educational distribution.

The measure of relative education ranges from 0 to 1 and allows for a straightforward interpretation. For example, an index of selectivity of 0.6 indicates that 60 percent of the population in the country of origin has acquired less or the same level of education as the individual migrant. In terms of the relative position in a distribution, we would also say that this person is positively selected, while a value below 0.5 would point to a negative selection.

### Additional Country of Origin Regional Data: Economic Conditions

In the second part of our study, we focus on the inaccuracy that is introduced when using country-level as opposed to regional-level data. We pursue this route to provide researchers with an idea of the extent of the inaccuracy for situations, in which regional information is not available. The inaccuracy is measured as the difference between two versions of relative education—one measured at the country level and one at the regional level.

The extent and the direction of the inaccuracy is likely related to regional disparities in educational opportunity structures and to regional outmigration. We expect the measurement inaccuracy to be more severe for countries, in which regional educational distributions differ from those of the overall country. For regions, in which the average education is considerably below the country mean, country-level measures will likely underestimate the extent of selectivity resulting in a “negative” inaccuracy. For illustration purposes, consider an individual who has acquired a secondary degree (ISCED 2) and resides in a region with subpar educational opportunities. Since only few of her peers will have completed a higher degree, her medium attainment will result in a higher score on relative education in that region. However, were we to compare her with the national average, where secondary education is the norm, she will score lower on relative education. The difference between these two measurements (i.e., the inaccuracy, which corresponds to subtracting the larger regional-level selectivity value from the smaller country-level value) will be negative. Conversely, we expect a “positive” inaccuracy (i.e., overestimating the extent of selectivity) for regions above the country mean, because in these contexts higher levels of absolute educational attainment will result in values of relative education that are more moderate. Note that in the absence of data on regional educational opportunity structures, we approximate these ideas by referring to the regional economic situation.

In addition, regional outmigration serves as a weighting factor, which does not exert a direct effect on the inaccuracy, but which is important as it can skew analyses of selectivity. For example, consider migrants from a region, in which the measurement inaccuracy is large. Assume in addition that individuals from this region rarely leave so that their outmigration rate is close the zero. The few individuals who do migrate are likely unproblematic in their contribution to the inaccuracy, as they do not show up in large enough numbers to distort analyses of the extent of educational selectivity.

Based on this reasoning, we consider (a) the economic opportunity structure at the regional level, (b) the economic opportunity structure at the country level, and (c) regional outmigration rates. While the measure of regional differences in economic opportunity structures capture within-country differences, the use of country-level measures allows accounting for potential additional cross-country patterns (e.g., the average inaccuracy may be higher in countries with high levels of within-country inequality in regional opportunity structures). The first measure (a) is based on regional information of the gross domestic product (GDP) and unemployment rates. Table 3 lists all data sources that were used for the different countries of origin. With the exception of Iraq, for which these measures were not available, we are able to include all countries. To build the second measure (b), we applied population weighted within-country standardization to make the information on GDP and unemployment rates comparable across origin countries. Subsequently, we calculated the population-weighted coefficient of variation (CV) as the standard deviation of a country's regional GDP (and unemployment rate) divided by the country's average GDP (and unemployment rate). Higher values on the CV of the GDP and the unemployment rate represent higher levels of regional inequality. The third measure (c) refers to differences in regional outmigration odds (ROO). They are measured using the ratios of relative regional outmigration based on our destination datasets and relative regional population based on our origin country datasets:

(1) $$\begin{array}{r} ROO=\frac{\frac{m_{ij}}{M_{i}}}{\frac{s_{ij}}{S_{i}}} \end{array}$$

where *m* refers to the number of emigrants from origin country *i* and its region *j, M* to the number of total emigrants from country *i, s* to the number of individuals living in country *i* and region *j* and *S* to the total population of country *i*. ROO values above 1 indicate higher outmigration from a specific region than expected assuming outmigration proportional to a region's size whereas values below 1 indicate the opposite. Values equal to 1 indicate that outmigration is exactly proportional to the region's size.

**Table 3:** Origin country data on regional GDP and unemployment rates.

**Country**	**Source**
Afghanistan^a^	https://openknowledge.worldbank.org/bitstream/handle/10986/27407/638720WP0Afgha00Box0361531B0PUBLIC0.pdf?sequence=1&isAllowed=y
Argentina	https://www.indec.gov.ar/nivel4_default.asp?id_tema_1=3&id_tema_2=9&id_tema_3=138 https://www.indec.gov.ar/nivel4_default.asp?id_tema_1=4&id_tema_2=31&id_tema_3=58
Bolivia	https://www.ine.gob.bo/index.php/producto-interno-bruto-departamental/producto-interno-bruto-departamental-5
Brazil	https://ww2.ibge.gov.br/estadosat/perfil.php?sigla=ro
Bulgaria	http://www.nsi.bg/en/content/5528/employed-persons-regions http://www.nsi.bg/en/content/5493/gdp-regions
Colombia	http://www.dane.gov.co/index.php/en/statistics-by-topic-1/regional-information/
Cuba^b^	http://www.one.cu/aec2011/esp/07_tabla_cuadro.htm
Ecuador	https://www.bce.fin.ec/index.php/component/k2/item/763
Morocco^a^	http://rgphencartes.hcp.ma/
Pakistan	https://en.wikipedia.org/wiki/List_of_Pakistani_provinces_by_gross_domestic_product http://www.pbs.gov.pk/sites/default/files/Labour%20Force/publications/lfs2013-14/t38-pak-fin.pdf
Peru	http://www.inei.gob.pe/estadisticas/indice-tematico/national-accounts/ http://www.inei.gob.pe/estadisticas/indice-tematico/ocupacion-y-vivienda/
Poland	https://geo.stat.gov.pl/imap/
Romania	http://edemos.insse.ro/portal/
Syria^c^	http://www.cbssyr.sy/index-EN.htm
Turkey	https://biruni.tuik.gov.tr/bolgeselistatistik/sorguSayfa.do?target=degisken
Ukraine	http://www.ukrstat.gov.ua/
Venezuela	http://www.ine.gov.ve/documentos/see/sistesisestadistica2008/estados/distritocapital/index.htm http://www.ine.gov.ve/index.php?option=com_content&view=category&id=103&Itemid=40

a *Regional GDP based on aggregated poverty rates*.

b *Regional unemployment data based on aggregated social expenditure*.

c *Regional GDP based on aggregated frequency data of incomebrackets*.

Finally, to analyze the inaccuracy, we construct a dataset where each row records the inaccuracy for each cross-classification of destination country, origin group, origin region, age, sex and educational attainment. In total, this data set comprises of 5,674 inaccuracy measurements for 23 origin group-destination country combinations (i.e., 18 origin groups of which four—Bulgarians, Moroccans, Poles, and Turks—are present in multiple destinations; see Table 1).

### Methods

In order to analyze the inaccuracy in measuring educational selectivity, we rely on a number of machine learning tools. In contrast to a conventional theory-driven approach, in which a set of hypothesized relationships is specified, the goal of this strategy is to account for as much of the inaccuracy as possible. Hence, we do not “impose” unnecessary restrictions on the data by a priori hypothesizing about how our constructs are related to the inaccuracy. Instead, we pass the data to a number of methods with the objective to model the data in such a way that it minimizes prediction error when given new data. This proceeding may be helpful when researchers are planning a study, which involves measures of educational selectivity. In these instances, it is important to know whether country-level measurements of educational distributions will suffice or whether regional measures are required. We train the algorithms on a training data set, which is based on an 80% random sample of the whole data set of inaccuracy measurements. The remaining data comprises the test set, which we use in the very end of the model-training phase to test the performance of the algorithms used.

**Figure 1:**
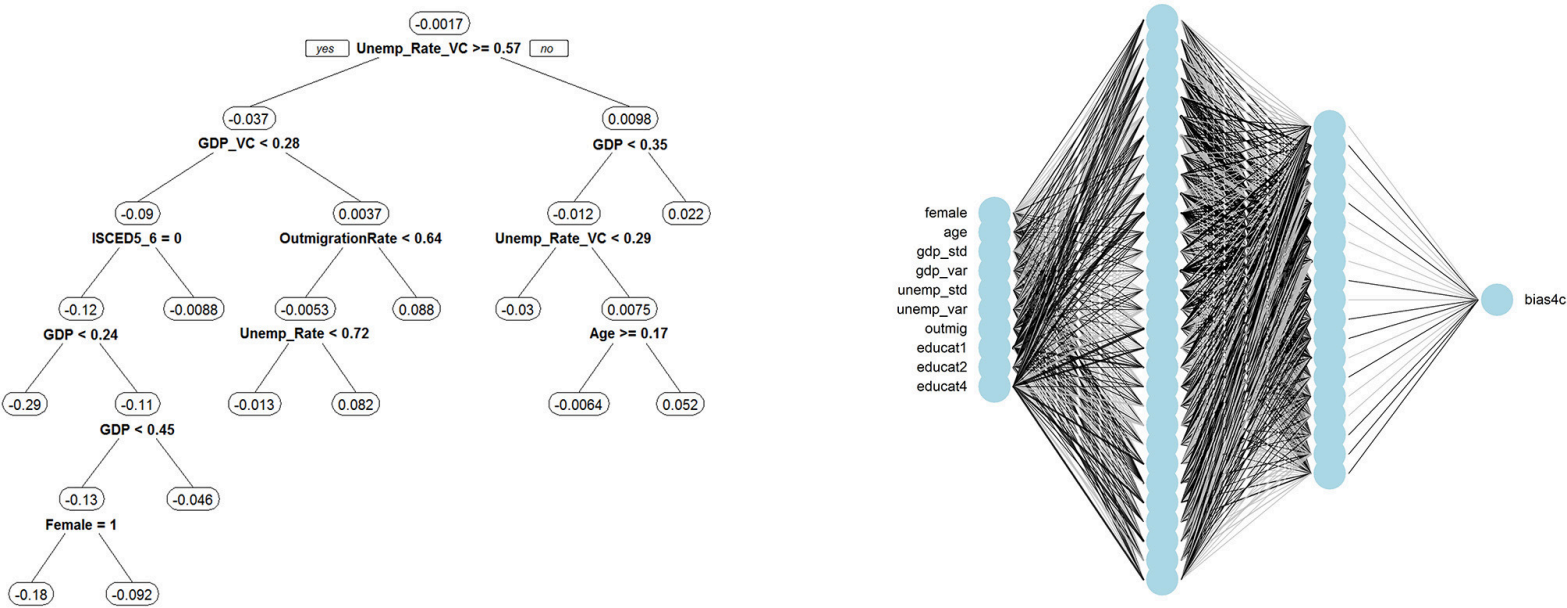
Visual presentation of selected machine-learning results. Regression tree and neural network. Black lines in the neural network connecting nodes indicate positive relationships whereas gray lines refer to negative relationships; the degree of line darkness indicates association strength.

We rely on three popular supervised learning methods to model the inaccuracy: random forests, the XGBoost algorithm and artificial neural nets. Random forests “grow” a large number of regression trees using random samples of the data and the predictors (Breiman, 2001). Random forests are a type of ensemble learning method, where the results of a larger number of “weak learners” are combined to obtain a better predictive performance than would be achievable by relying on a weak learner alone. In the case of random forests, a regression tree represents the weak learning method. Each regression tree creates root nodes that split the data into disjunctive groups based on values of the predictor variables. Variables used to split nodes closer to the root of the tree are more important than variables used to split further away where importance is defined as the largest decrease in the residual sum of squares. The panel on the left-hand side of Figure 1 presents the result of one such tree: for this specific tree, GDP is at the root of the regression tree splitting the data into those regions that score above (right branch) and below (left branch) values of 0.3. Following the right branch would help us understand comparatively small positive inaccuracy, whereas following the left branch would do the same for negative inaccuracy values. For example, we are likely to encounter a substantial average inaccuracy of 0.17 for immigrants from regions with a GDP of equal to or above 0.4 (i.e., following the right branch: GDP < 0.3? No! -> GDP < 0.4? No! -> GDP> = 0.4? No! -> inaccuracy = 0.17). In total, our random forest grows 500 of these trees and combines their estimates to make predictions.

The XGBoost algorithm represents a variation of the random forests idea. Whereas, random forests are a type of “bagging” method where regression trees are estimated effectively in parallel and combined at the end, XGBoost is an example for a tree-based “boosting” method (Chen and Guestrin, 2016). Boosting methods iteratively learn from “mistakes” by specifically focusing on reducing the residual error from the previous estimation step (i.e., the previously estimated regression tree).

Lastly, artificial neural nets are typically referred to as “black box” methods where a set of inputs passes through a series of hidden layers to predict the output (Goodfellow et al., 2016). The panel on the right-hand side of Figure 1 provides a visual representation of the artificial neural network used in this study. On the left, the input layers appear. Every input is connected to all nodes of the first hidden layer by a set of weights. In essence, every node represents a regression equation whose output passes through an activation function before being “passed along the network.” A hidden layer can thus be thought of as stacked regression models. The first hidden layer is also connected to all nodes of the second hidden layer, which is connected to the output layer predicting the inaccuracy. This forward pass through the network is used in the so-called backpropagation step to adjust iteratively the weights connecting the various layers in order to minimize prediction error in the inaccuracy measures.

Each method has a certain set of hyperparameters that can be tuned to improve model performance (e.g., the number of trees grown in a random forest or the number of hidden layers and their nodes in artificial neural nets). We thus drew another 30 percent sample from the training set to serve as a cross-validation test set for hyperparameter tuning using grid-search methods (Goodfellow et al., 2016; Géron, 2017). All models and an extensive tutorial, which provides information on how to use them to predict the inaccuracy using new data is made freely available to researchers on the first author's GitHub (https://github.com/chspoerlein/selectivity_tutorial).

## Results

### Describing Educational Selectivity

**Figure 2:**
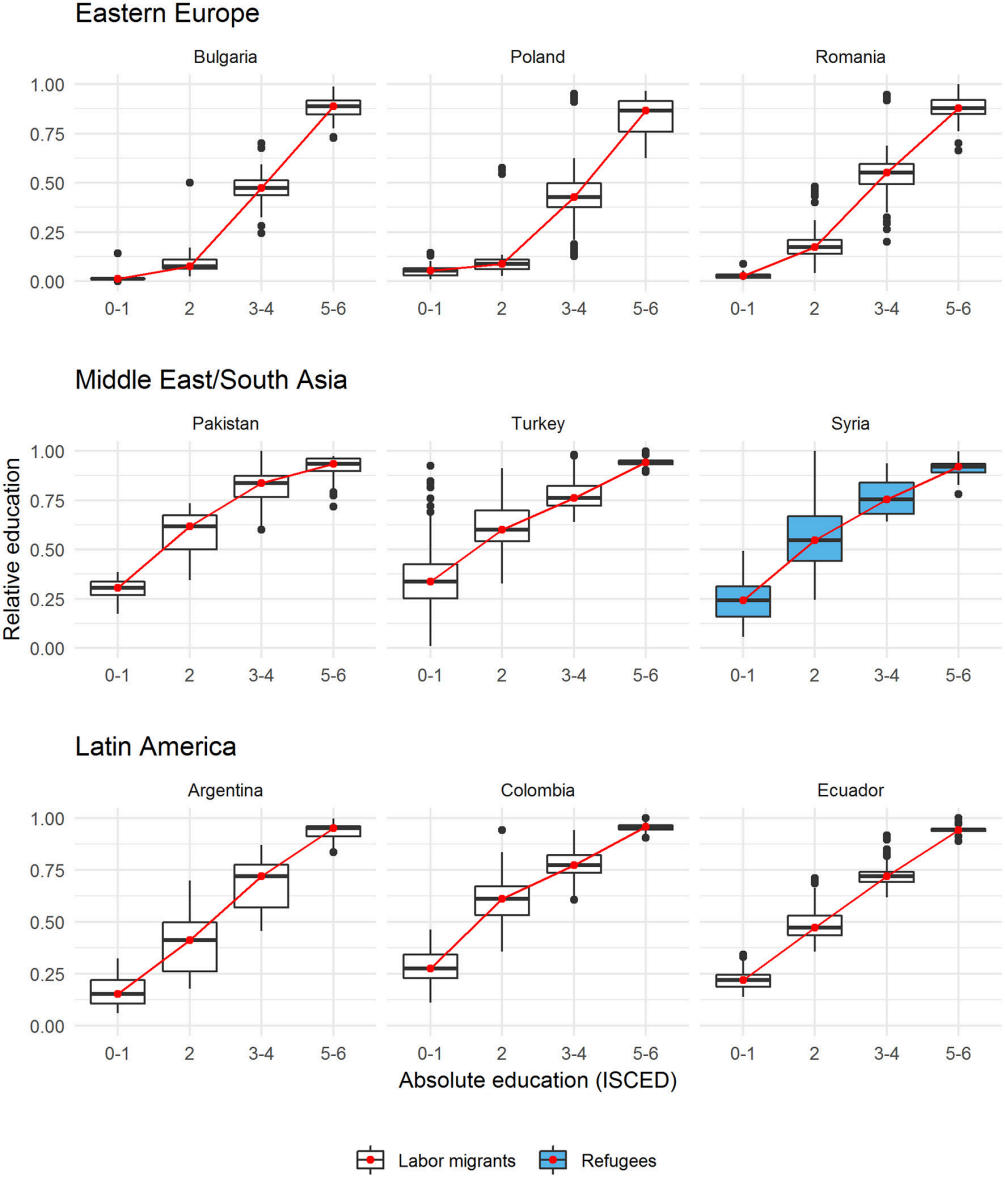
Relative and absolute education.

Before presenting the main findings, we first illustrate the relationship between absolute and relative education. Figure 2 depicts boxplots of the distributions of relative education for each category of absolute education and for a subset of the three numerically largest origin groups from each geographic region (with the exception of Africa; the full set of boxplots for all migrant groups is included in Figure S1).

For each origin group, the medians are connected with a red line to visualize the general relationship. Unsurprisingly, absolute and relative education are strongly positively correlated (*r* ~ 0.81) with individuals in the higher ISCED categories also scoring higher on the measure of relative education. The key aspect depicted in Figure 2 relates to the variation of relative education *within* each category of absolute education. That is, the scores on relative education are highly variable, especially among the mid-level educational categories (ISCED 2 and 3–4). Syrian refugees with ISCED 2 represent a good example for this phenomenon. Their median relative education is at around 0.60 but the box (i.e., the interquartile range) covers a 25-point interval ranging from around 0.45 to 0.70. In other words, the value or meaning of education is context-depended: individuals with nominally the same (medium) degree find themselves in very different positions in their origin region's educational hierarchy. Among labor migrants, individuals from Argentina with ISCED 2 represent a similarly impressive example with a median of 0.40 and an interquartile range, which spans a 25-point interval from 0.25 to 0.50. In general, an individual's position in the origin region's educational hierarchy is considerable more variable for the mid-level educational categories opposed to the lowest and highest categories.

**Figure 3:**
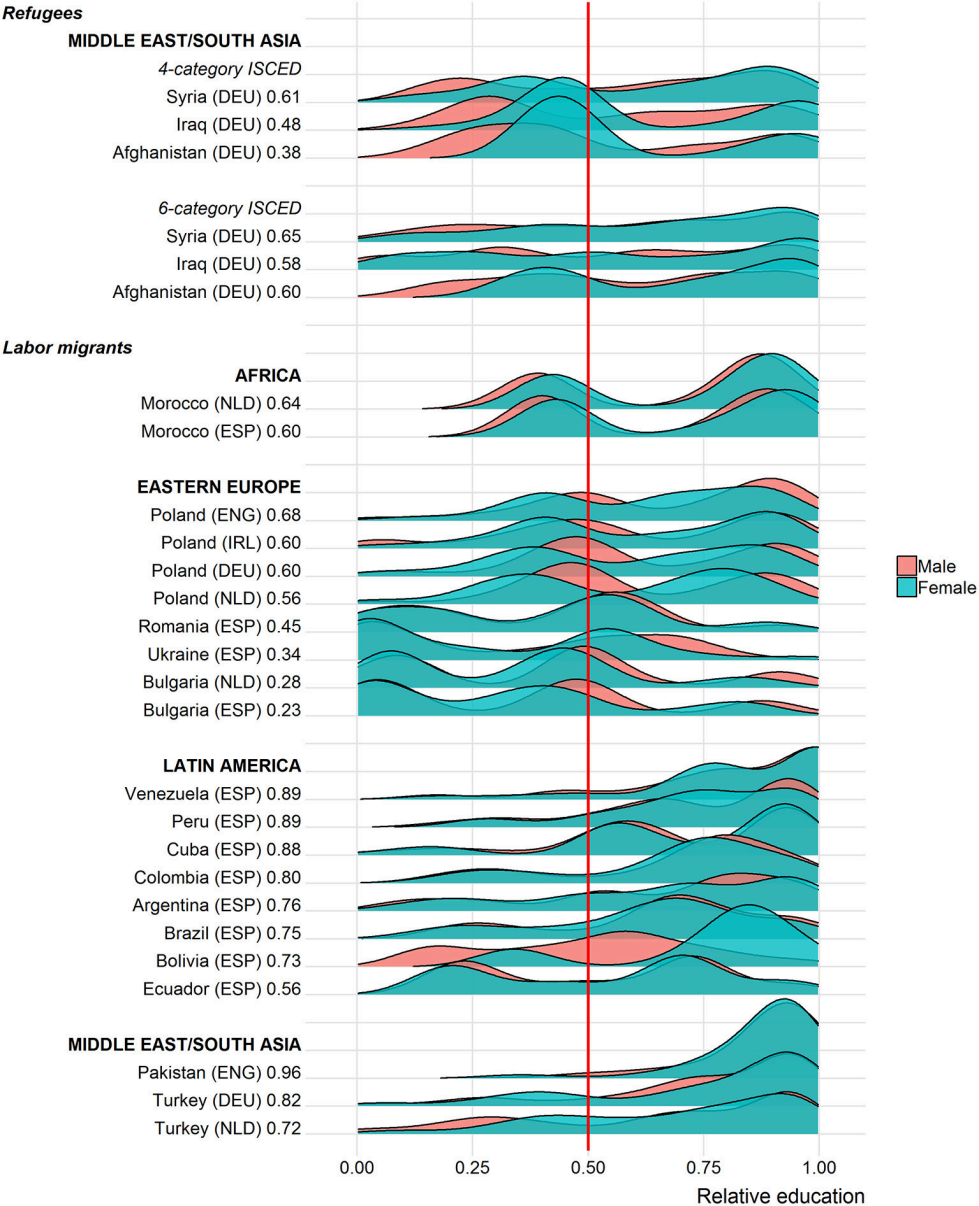
Gender differences in educational selectivity. DEU, Germany; ENG, England; ESP, Spain; IRL, Ireland; NLD, Netherlands.

Figure 3 presents the selectivity profiles separately for labor migrants and refugees^3^. It also covers gender differences. Each row represents one origin group-destination country combination and their respective density distribution of relative education (x-axis) grouped into geographic regions. The red reference line indicates the 0.5 threshold. In distributional terms, we would say that individuals above this threshold are positively selected, while immigrants below this threshold are negatively selected. Within the group of labor migrants and refugees as well as within each geographic region, origin groups are ranked according to the percentage of positively selected immigrants. This share is also included in the numbers following the indication of the origin group-destination country combination. Note that due to differences in the measurement of educational attainment discussed in The Selectivity Measure: Relative Education, the profiles for refugees are presented using the four-category ISCED variant, which is also applied to labor migrants, and in addition using a more detailed six-category ISCED variant that is only available for refugees. The discussion of results in the text is based on the four-category ISCED specification if not stated otherwise.

Overall, Pakistani immigrants in England are the most positively selected group among labor migrants (with 96 percent above the threshold of 0.5), whereas Bulgarian immigrants in Spain are the least positively selected group (with 23%). On average and across all origin groups and destination countries in this study, immigrants score 0.61 on our measure of regional educational selectivity suggesting that their educational attainment is at least as high as that of roughly 60 percent of the sedentary population of the same sex and age group who remained in the migrant's region of origin.

One major point is immediately apparent from Figure 3. That is, each origin group is composed of varying shares of both positively and negatively selected individuals covering the whole spectrum of educational selectivity. Only in a few cases, is it reasonable to characterize a whole origin group (e.g., more than 90 percent) as positively or negatively selected. In this study, only recent immigrants to England from Pakistan would represent such an extreme case. For all other groups, the distributions of the specific proportions vary substantially. For some labor migrant groups, a large majority (>80%) crosses the 0.5 threshold. These groups include Columbians, Cubans, Peruvians, and Venezuelans in Spain, Turks in Germany and Pakistanis in England. In contrast, Eastern Europeans (with the exception of Poles) are located mostly below the threshold. In addition, many origin groups show distinctly bimodal distributions, according to which a substantive share of the group is negatively selected while another substantive share is positively selected (e.g., Moroccans in Spain and the Netherlands, Polish migrants in all three destinations or Ecuadorians in Spain). Moreover, there are no clear patterns visible when it comes to geographic origins. Irrespective of whether immigrants originate from Africa, Eastern Europe, the Middle East, Latin America or South Asia, they seem to cover a wide array of positively and negatively selected individuals.

Turning to the selectivity profiles of refugee migrants in Germany, we present two descriptions. The first relies on the less detailed measure of educational attainment that was also used for labor migrants, while the second description is based on the more detailed variant of the measure of educational attainment that was only available for refugees (see The Selectivity Measure: Relative Education). The comparison illustrates that rather divergent assessments of the selectivity profiles can result from different specifications. Relying on the less detailed 4-category ISCED variant suggests that 62 percent of the Afghan refugees are located below the 0.5 threshold indicating that this group is mostly negatively selected. Among Iraqis, about half of the migrants are positively selected (48 percent); for Syrians, this share amounts to 61 percent. With the 6-category ISCED measure, little changes for Syrian refugees: still, about two-thirds of them are positively selected (65 percent). However, both Iraqis (with 58 percent) and especially Afghan refugees (with 60 percent) shift toward the positive end of the selectivity spectrum. Apparently, collapsing ISCED 0 and 1 and ISCED 3 and 4 into single categories distorts the descriptions of selectivity for these groups. For Afghan and Iraqi refugees, the distinction between having no education and having completed primary education appears essential. Merging the two lowest ISCED categories into one category thus conceals the underlying positive selectivity inherent in this refugee migration. At the same time, in Western societies, the lowest ISCED categories are populated by so few adults that these categories are virtually meaningless. Accordingly, the more detailed variant of the ISCED classification, which distinguishes between ISCED 0 and 1, depicts a more positive take on selectivity than when considering the less informative four-category ISCED specification.

What do these findings imply for the comparison between refugees and labor migrants? The most important message seems to be that differences in relative education are comparatively small. They are certainly less pronounced than arguments in the literature suggest. The overall group means for the two populations amount to 0.59 for refugees (0.61 using the 6-category education measure) and to 0.62 for labor migrants. Moreover, a closer look at the findings reveals substantive variation in this difference across groups. In fact, labor migrants of certain origins score similar or lower on the measure of educational selectivity than do the refugees covered in this study. These are mostly immigrants from Eastern Europe (i.e., Bulgarians in the Netherlands and Spain as well as Ukrainians and Romanians in Spain). At the same time, there is more variation within origin groups than there is across origin groups—irrespective of the migration motive. This result underlines our initial point that all groups are composed of varying portions of negatively and positively selected individuals.

As the refugee data only covers Germany, contrasting refugees to Germany with labor migrants to a broader set of destination countries may not be the most insightful comparison. Focusing only on labor migrants to Germany, however, restricts the description to recent Polish and Turkish migrants. This comparison leads to the same conclusion with the levels of selectivity being broadly similar to those of refugees in both cases (Poles: 0.61 and Turks: 0.75).

Figure 3 also plots gender differences in educational selectivity. On average, there is virtually no difference between male and female migrants (0.62 vs. 0.61) across all groups. Nevertheless, for some groups distinct patterns emerge. Female refugees score higher on the selectivity scale than their male counterparts irrespective of the ISCED specification (about 5 points among Syrians, 6 points among Afghans and 4 points among Iraqis). It should be kept in mind, however, that the share of males among refugees in the peak year 2015, in which the largest number of refugees came to Germany, has been with about 70 percent much higher than that of women (Bundesamt für Migration und Flüchtlinge., 2016). For labor migrants only one case stands out: female Bolivians who are considerably more positively selected compared to their male counterparts (+25 points).

### Addressing Measurement Inaccuracy

**Figure 4:**
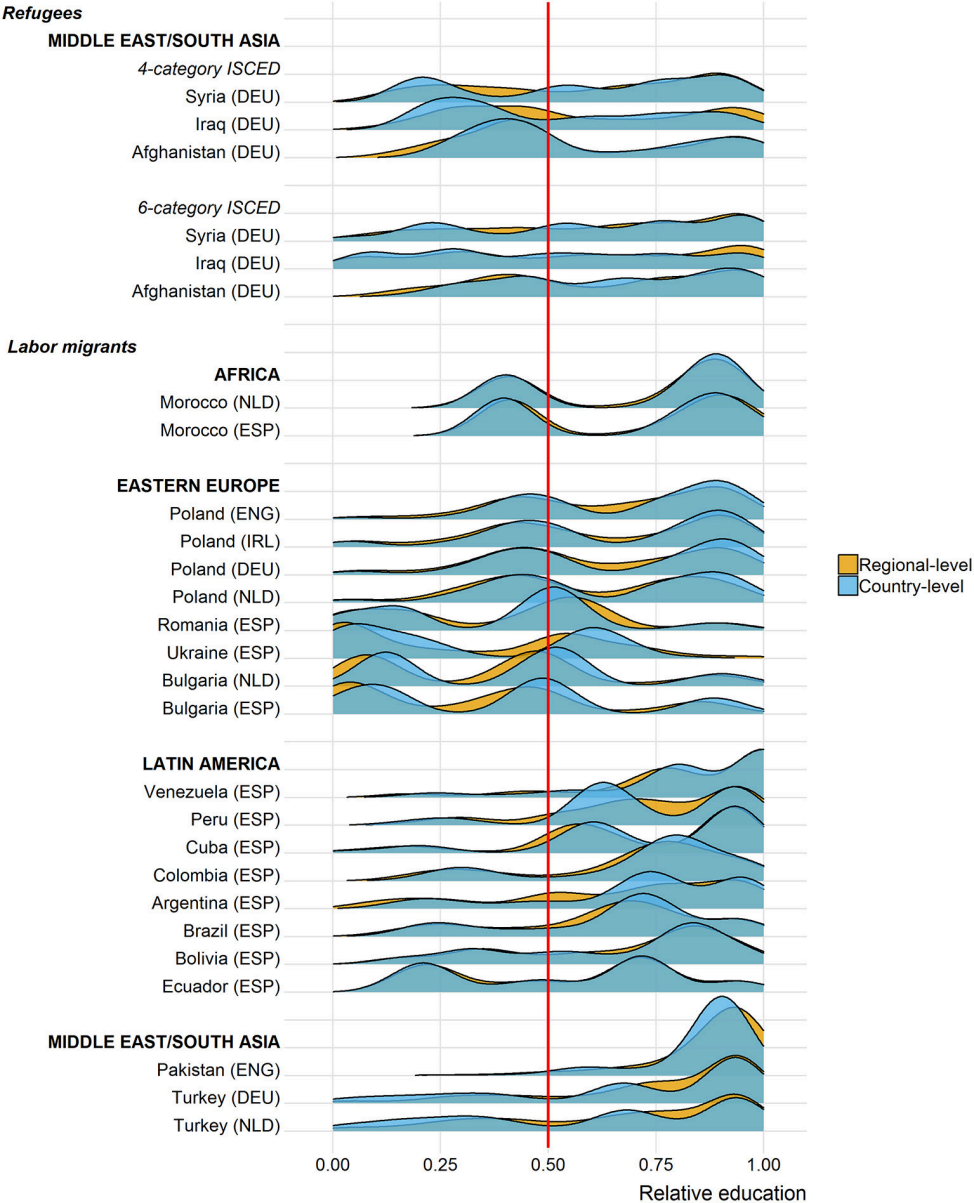
Educational selectivity measured at the regional level vs. the country level. DEU, Germany; ENG, England; ESP, Spain; IRL, Ireland; NLD, Netherlands.

Up to now, we presented educational selectivity profiles based on the regionalized reference distributions. Figure 4 illustrates the same regionalized profiles and, in addition, depicts the selectivity distributions based on country-level information. This aggregate comparison allows for a first assessment of the measurement inaccuracy that is introduced when using educational distributions at the country level as opposed to the regional level. In general, the more strongly the two distributions overlap, the less severe the measurement inaccuracy is and the lower is the additional benefit of collecting regional information. Overall, there is no clear pattern in terms of one measurement approach consistently leading to over- or underestimating immigrant selectivity. Rather than that, there tend to be few differences. For some origin groups, there are discrepancies in the distributional overlap. Visually, this is noticeable for Iraqis where we see higher levels of relative education at the regional compared to the country level. A similar though less pronounced pattern is present for Syrians, whereas the opposite pattern is found for Bulgarians.

**Figure 5:**
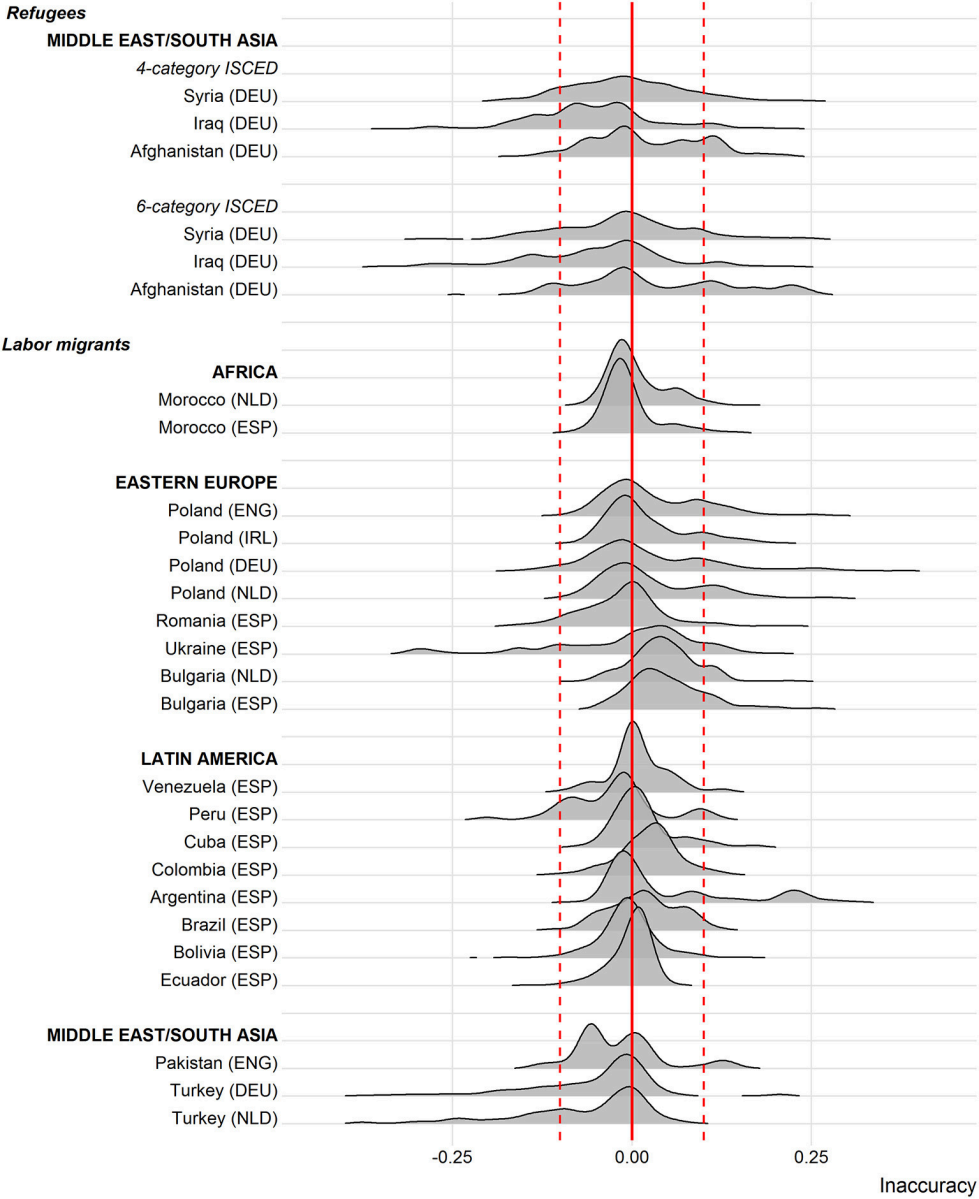
Differences between educational selectivity measured at the regional level vs. the country level. DEU, Germany; ENG, England; ESP, Spain; IRL, Ireland; NLD, Netherlands.

Figure 5 captures the inaccuracy between the two approaches to measuring selectivity in a direct manner by subtracting the regional measure from the country-level measure (x-axis). Values close to the red reference line at zero reflect little to no differences in the two measures. Portions of the distribution to the right of the red reference line indicate that educational selectivity measured regionally leads to larger estimates of relative education than if measured at the country level, while the opposite is true for values to the left of the red line. Overall, measurement inaccuracy is an issue for all immigrant groups considered here. At the same time, in only a few origin groups, the inaccuracy goes overwhelmingly in one direction indicating a systematic over- or underestimation of educational selectivity. More importantly, in most cases the inaccuracy remains within limited ranges with only a small proportion of cases exceeding inaccuracy levels of 0.1 (represented by the dashed red lines).

The Argentinian origin group is a case that deserves attention. Here, a substantial portion of the distribution takes values above 0.20 suggesting that relying on country-level data would overestimate educational selectivity by more than 20 points. A closer look at the data reveals that these immigrants mostly stem from only a few regions, which happen to be inadequately characterized by the country average educational distribution. That is, roughly two-thirds of Argentinian migrants originate from Buenos Aires and the surrounding province. In this urban context, acquiring a higher educational degree is more common than in other regions of Argentina. Comparing emigrants from Buenos Aires to the average Argentinian, therefore, makes them seem more positively selected than they actually are when compared to their “real peers” in Buenos Aires. Argentina provides an extreme example for a country, in which disproportionate outmigration from certain regions coincides with substantive differences between the educational distributions typical for these regions as opposed to the whole country.

A similar situation, though this time in the opposite direction, is present for Turks and to some degree also for Ukrainians. In line with the Argentinian case, outmigration in these countries is more pronounced in certain regions than in others. In contrast to migration flows from a highly developed region in Argentina, Turkish and Ukrainian migrants tend to emigrate from a limited number of regions, which score well below the national average. Accordingly, what in the national context would be considered as a medium level of education is more valuable in lower developed regions, in which relatively fewer individuals complete such a degree. The use of country averages in these instances yields an underestimate of the “true” extent of selectivity.

Finally, our aim is to generate tools that help researchers to identify origin groups, for which the expected inaccuracy in measuring educational selectivity reaches unacceptable levels. This is achieved by modeling the relationship between regional inaccuracy levels and characteristics of these regions and countries using random forests, the XGBoost algorithm and neural nets. Table 4 reports the results as forecasting errors of testing our trained methods on the 20 percent holdout sample. It includes the mean absolute error (MAE), which measures the average magnitude of the prediction error, and the root mean squared error (RMSE). Note that both fit measures are scale-dependent. According to Table 4, the random forest and XGBoost performed best with MAEs of 0.018 and 0.015, respectively. The prediction error associated with the neural net is more than twice as large. More concretely, were we to use either of the two tree-based methods to predict the inaccuracy in selectivity in future research projects, we would expect the average prediction to be off by 0.018 and 0.015 points. Considering the inaccuracy distributions captured in Figure 5 and an arbitrarily set limit of defining an acceptable inaccuracy as within 0.1 points, we are able to predict the expected inaccuracy quite accurately. Tree-based methods are also preferable when relying on RMSE estimates, which penalize large errors.

**Table 4:** Machine-learning techniques to minimize the inaccuracy in selectivity measures (country-level relative education minus regional-level relative education).

**Model**	**Mean absolute error (MAE)**	**Root mean square error (RMSE)**
Random forest	0.014	0.026
XGBoost	0.015	0.030
Neural net	0.035	0.079

## Conclusions

In this descriptive piece, we illustrated the selectivity profiles of a range of immigrant groups who arrived in Western Europe in recent years. We focused on refugees from conflict regions in the Middle East and South Asia and contrasted them to labor migrants from a wide variety of origins. By comparing refugees to labor migrants, we addressed longstanding assumptions about the selectivity patterns dominant among migrants who flee from their home country vs. migrants who leave for economic reasons. For this undertaking, we built upon prior approaches to measuring selectivity. Our individual-level measure of selectivity identified each migrant's relative position in the age- and gender-specific educational distribution of the country of origin. We further refined this approach by considering immigrants' regional origins and, accordingly, constructed the educational distributions present in the origin countries for each region as opposed to the whole country.

One of the key findings is that migrant groups are almost never either positively or negatively selected, but are composed of varying proportions of both positively and negatively selected individuals. In other words, even though a group may be heavily positively selected in that the majority of its members score above 0.5, there is usually also a considerable proportion of that origin group that is negatively selected.

A second key result is that there are few differences between refugees and labor migrants in average levels of selectivity. However, these differences vary; and there are labor migrant groups that score similar or lower on educational selectivity than do the refugee groups covered in this study. In other words, the differences between these two populations who migrate for different reasons is considerably less prominent than arguments in the literature seem to suggest.

Finally, regional origins matter—though not universally. Our findings show that there are cases where considering educational distributions at the country level rather than at the regional level produces a considerable inaccuracy in the measure of selectivity. This inaccuracy is likely to occur in countries where outmigration is confined to particular regions of the country and where these regions exhibit economic opportunity structures that are either substantially below or above the country average. In these instances, the positioning of the individual migrant in the educational distribution of the country as a whole produces an inadequate assessment of a person's relative position. Thus, depending on the research interest it might be reasonable to make the effort and check whether it is possible to include a regional measurement of selectivity. If this is not feasible, researchers may use the pre-trained machine-learning tools that are made freely available to get an idea about the direction and the size of the inaccuracy.

Challenging for any approach, which includes a wide range of immigrant groups of different origins in different destinations, are the obstacles inherent to using a variety of data sources. Our description is limited in that we cannot claim to come up with a representative or fully comparable empirical account across the migrant groups and destinations included in our study. The destination country data sets cover slightly different periods of immigration and they used different sampling strategies. Hurdles of this kind will be difficult to overcome especially for an extremely mobile population of recently arrived migrants who move a lot in the first months and years after arrival and for whom sampling frames in many destinations are not available. The origin country data do not provide a uniform source of information either, but vary in the number of cases included, in quality and in how recently they were collected. Harmonization issues further complicate the picture. To assign each migrant to the appropriate age- and gender-specific educational distribution in the region or country of origin, it is necessary to aggregate the ISCED categories. Otherwise, it is not possible to analyze destination country data together with origin country data. The lowest ISCED categories (0 and 1) had to be summarized because most destination countries do not collect information on the zero category (which they consider not to be existent in their countries). The medium categories (ISCED 3 and 4) were analyzed together, because vocational training (i.e., ISCED 4) is not present in all countries. The highest categories (ISCED 5 and 6) are summarized because only few individuals complete a doctoral degree (i.e., ISCED 6) and because not all countries of origin collect information on this highest category. Harmonization of this kind, obviously, reduces the degree of precision in descriptions of educational selectivity. The differences in the selectivity profiles of refugees we saw when using different categorizations of educational attainment attest to this concern.

The selection of countries constitutes another limitation. It was driven by pragmatic reasons, as information on migrants' regional origin has been available only for the three destination data sources considered in this study. Surely, a selection of origin and destination countries based on substantive reasons would be preferable. With regard to destinations, key immigration countries in Western Europe are missing (e.g., France, Italy, or Sweden). Regarding the countries of origin, only few African and Asian countries are present in our description.

Educational selectivity is an important characteristic that has been shown to be relevant for immigrants' and their children's incorporation into the host societies (e.g., Ichou, 2014; Spörlein and Kristen, 2018). Similarly, other selectivity characteristics such as motivational traits or attitudes may matter for how well migrants fare in the years after arrival, possibly also in the next generation. However, it will be even more difficult to come up with suitable data on such attributes for migrants in the destination countries and for the populations in the origin countries.

## Author Contributions

All authors listed have made a substantial, direct and intellectual contribution to the work, and approved it for publication.

### Conflict of Interest Statement

The authors declare that the research was conducted in the absence of any commercial or financial relationships that could be construed as a potential conflict of interest.

## Footnotes

^1^The Ukraine is not a member state of the EU, but belongs to Eastern Europe geographically.

^2^The account on measuring educational selectivity in this section as well as in section Measuring Educational Selectivity is based on an earlier presentation of our reasoning (see Spörlein and Kristen, 2018).

^3^Figure S2 in the Supplementary Material presents these findings ordered according to the distributions rather than according to geographic regions.
