# Supplementary material for: Why We Should Care About Regional Origins: Educational Selectivity Among Refugees and Labor Migrants in Western Europe
Source: Front Sociol. 2019 May 7;4:39. doi: 10.3389/fsoc.2019.00039 (PMC8022669; doi:10.3389/fsoc.2019.00039)
Supplement: Supplementary file 1 [file Presentation_1.pdf]

Figure S1: Relative and absolute education for all origin groups

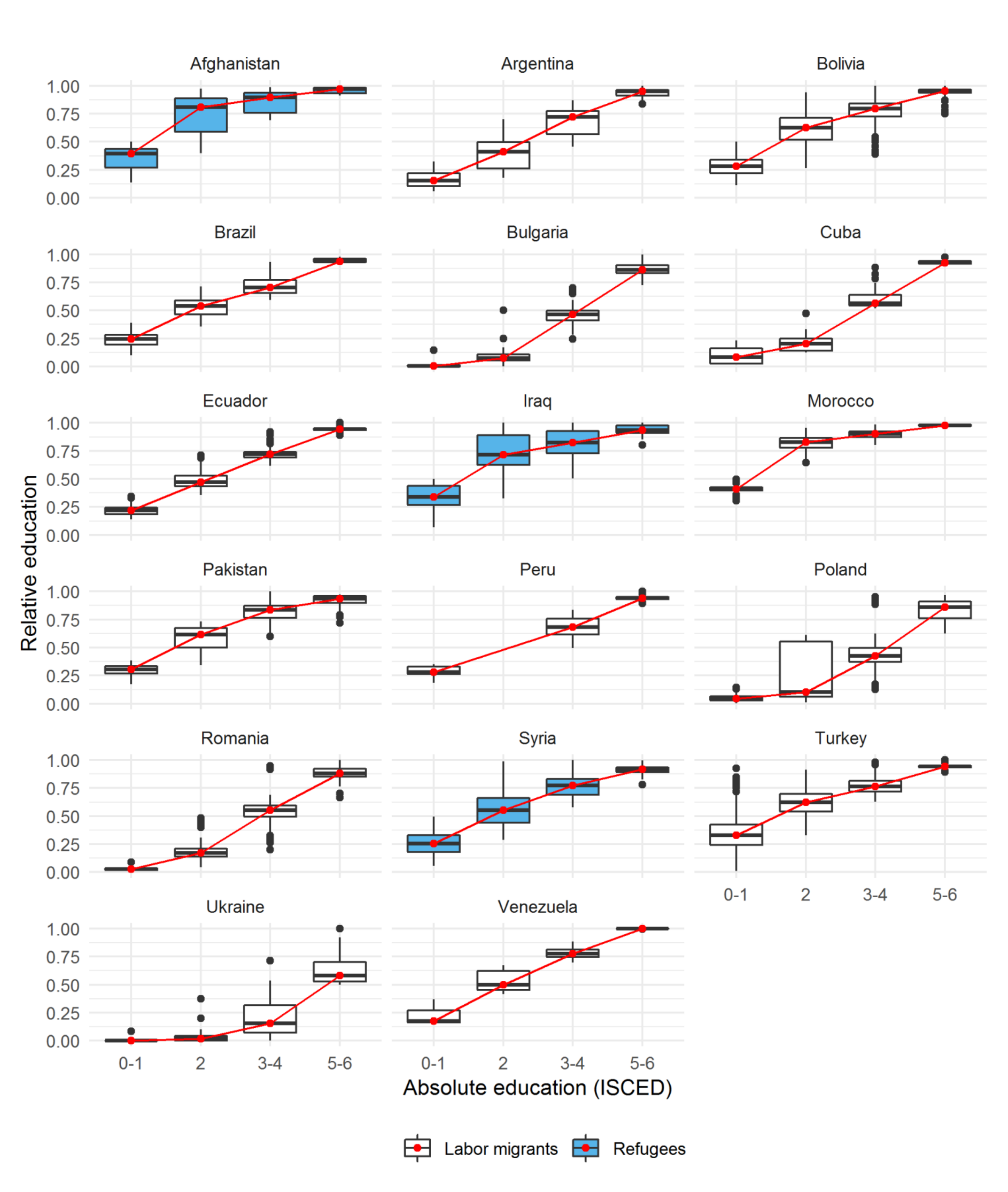

Figure S2: Alternative illustration of gender differences in educational selectivity

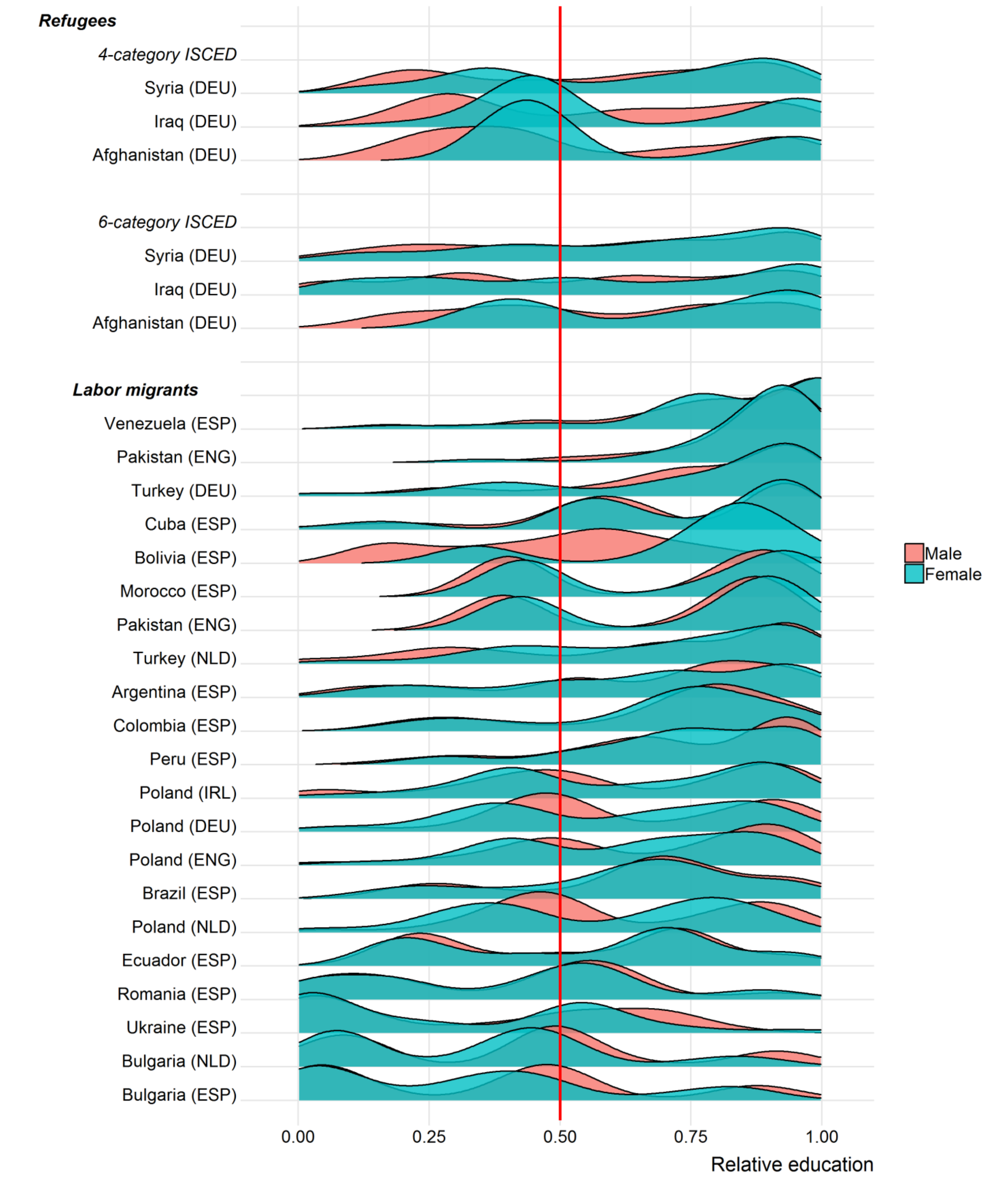

Note: DEU=Germany; ENG=England; ESP=Spain; IRL=Ireland; NLD=Netherlands.
