# Supplementary material for: Why We Should Care About Regional Origins: Educational Selectivity Among Refugees and Labor Migrants in Western Europe
Source: Front Sociol. 2019 May 7;4:39. doi: 10.3389/fsoc.2019.00039 (PMC8022669; doi:10.3389/fsoc.2019.00039)
Supplement: Supplementary file 2 [file Data_Sheet_1.docx]

# Supplementary Material

Table S1: Distributions for labor migrants according to country of origin

|  |  | **Africa** | | **Eastern Europe** | | | | | | | | **Latin America** | | | | | |
| --- | --- | --- | --- | --- | --- | --- | --- | --- | --- | --- | --- | --- | --- | --- | --- | --- | --- |
|  |  | Morocco | | Bulgaria | | Poland | | Romania | | Ukraine | | Argentina | | Bolivia | | Brazil | |
|  |  | N | % | N | % | N | % | N | % | N | % | N | % | N | % | N | % |
| Gender |  |  |  |  |  |  |  |  |  |  |  |  |  |  |  |  |  |
|  | Male | 370 | 59 | 271 | 47 | 1621 | 52 | 518 | 47 | 64 | 39 | 180 | 46 | 107 | 36 | 53 | 34 |
|  | Female | 255 | 41 | 304 | 53 | 1481 | 48 | 591 | 53 | 99 | 61 | 209 | 54 | 188 | 64 | 105 | 66 |
| Absolute education | |  |  |  |  |  |  |  |  |  |  |  |  |  |  |  |  |
|  | ISCED 0,1 | 241 | 39 | 46 | 8 | 80 | 3 | 172 | 16 | 14 | 9 | 52 | 13 | 60 | 20 | 29 | 18 |
|  | ISCED 2 | 113 | 18 | 178 | 31 | 66 | 2 | 280 | 25 | 31 | 19 | 45 | 12 | 38 | 13 | 25 | 16 |
|  | ISCED 3,4 | 236 | 38 | 261 | 35 | 1389 | 45 | 567 | 51 | 54 | 33 | 172 | 44 | 152 | 52 | 79 | 50 |
|  | ISCED 5,6 | 35 | 6 | 90 | 16 | 1570 | 51 | 90 | 8 | 64 | 39 | 120 | 31 | 45 | 15 | 25 | 16 |
| Relative education | | Mean | Median | Mean | Median | Mean | Median | Mean | Median | Mean | Median | Mean | Median | Mean | Median | Mean | Median |
| (regional) |  | 0.70 | 0.83 | 0.37 | 0.41 | 0.62 | 0.65 | 0.40 | 0.48 | 0.32 | 0.25 | 0.66 | 0.73 | 0.67 | 0.77 | 0.64 | 0.68 |
|  |  |  | |  |  |  |  |  |  |  |  |  | | |  |  |  |
|  |  | **Latin America** | | | | | | | | | | **Middle East/South Asia** | | | |  |  |
|  |  | Colombia | | Cuba | | Ecuador | | Peru | | Venezuela | | Pakistan | | Turkey | |  |  |
|  |  | N | % | N | % | N | % | N | % | N | % | N | % | N | % |  |  |
| Gender |  |  |  |  |  |  |  |  |  |  |  |  |  |  |  |  |  |
|  | Male | 254 | 40 | 43 | 43 | 439 | 47 | 61 | 44 | 47 | 42 | 508 | 80 | 820 | 53 |  |  |
|  | Female | 387 | 60 | 57 | 57 | 493 | 53 | 78 | 56 | 66 | 58 | 127 | 20 | 723 | 47 |  |  |
| Absolute education | |  |  |  |  |  |  |  |  |  |  |  |  |  |  |  |  |
|  | ISCED 0,1 | 113 | 18 | 4 | 4 | 308 | 33 | 14 | 10 | 6 | 5 | 6 | 1 | 352 | 23 |  |  |
|  | ISCED 2 | 86 | 13 | 8 | 8 | 154 | 17 | 0 | 0 | 12 | 11 | 62 | 10 | 163 | 11 |  |  |
|  | ISCED 3,4 | 341 | 53 | 41 | 41 | 374 | 40 | 70 | 50 | 41 | 36 | 40 | 6 | 394 | 26 |  |  |
|  | ISCED 5,6 | 101 | 16 | 47 | 47 | 96 | 10 | 55 | 40 | 54 | 48 | 527 | 83 | 634 | 41 |  |  |
| Relative education | | Mean | Median | Mean | Median | Mean | Median | Mean | Median | Mean | Median | Mean | Median | Mean | Median |  |  |
| (regional) |  | 0.69 | 0.76 | 0.71 | 0.76 | 0.54 | 0.67 | 0.74 | 0.79 | 0.83 | 0.87 | 0.87 | 0.92 | 0.73 | 0.80 |  |  |

Table S2: Distributions for refugees according to country of origin

|  |  | **Middle East/South Asia** | | | | | |
| --- | --- | --- | --- | --- | --- | --- | --- |
|  |  | Afghanistan | | Iraq | | Syria | |
|  |  | N | % | N | % | N | % |
| Gender |  |  |  |  |  |  |  |
|  | Male | 286 | 62 | 310 | 64 | 1299 | 63 |
|  | Female | 174 | 38 | 175 | 36 | 747 | 37 |
| Absolute education | |  |  |  |  |  |  |
|  | ISCED 0,1 | 282 | 61 | 246 | 51 | 638 | 31 |
|  | ISCED 2 | 63 | 14 | 102 | 21 | 441 | 22 |
|  | ISCED 3,4 | 72 | 16 | 66 | 14 | 479 | 23 |
|  | ISCED 5,6 | 43 | 9 | 71 | 15 | 488 | 24 |
| Relative education | | Mean | Median | Mean | Median | Mean | Median |
| (regional) | | 0.55 | 0.46 | 0.57 | 0.47 | 0.60 | 0.66 |
|  | | N | % | N | % | N | % |
| Absolute education | |  |  |  |  |  |  |
|  | ISCED 0 | 127 | 28 | 97 | 20 | 122 | 6 |
|  | ISCED 1 | 155 | 34 | 149 | 31 | 516 | 25 |
|  | ISCED 2 | 63 | 14 | 102 | 21 | 441 | 22 |
|  | ISCED 3 | 63 | 14 | 57 | 12 | 430 | 21 |
|  | ISCED 4 | 9 | 2 | 9 | 2 | 49 | 2 |
|  | ISCED 5,6 | 43 | 9 | 71 | 15 | 488 | 24 |
| Relative education | | Mean | Median | Mean | Median | Mean | Median |
| (regional) | | 0.64 | 0.71 | 0.56 | 0.58 | 0.62 | 0.67 |
